# Clinical Profile and Psychosocial Correlates of Childhood Depressive Disorder: A Case-Control Study

**DOI:** 10.7759/cureus.107267

**Published:** 2026-04-17

**Authors:** Pallavi Priyam, Uday Sankar Mandal, Abhishek Dandapath, Saswati Nath

**Affiliations:** 1 Department of Psychiatry, R. G. Kar Medical College and Hospital, Kolkata, IND; 2 Department of Psychiatry, Bankura Sammilani Medical College and Hospital, Bankura, IND; 3 Department of Psychiatry, Malda Medical College and Hospital, Malda, IND

**Keywords:** childhood depression, family environment, parent-child perceptual discordance, psychosocial correlates, temperament

## Abstract

Introduction: Historically, childhood depression was overlooked due to perceived developmental immaturity. However, modern paradigms recognize it as a complex disorder often presenting with "masked" symptoms that differ from adult manifestations. In India, prevalence rates show an increasing trend, yet research on younger children remains sparse. This study aimed to delineate the clinical features, temperamental traits, and family environmental correlates of childhood depressive episodes in an eastern Indian hospital setting, while quantifying the perceptual gap between children and parents.

Methods: This institution-based, case-control study screened 145 children aged 5-12 years, resulting in a final population of 34 cases (diagnosed via the International Classification of Diseases, 10th Revision, Diagnostic Criteria for Research) and 34 age- and sex-matched controls. Assessment tools included the Pediatric Symptom Checklist (PSC), Center for Epidemiological Studies Depression Scale for Children (CES-DC), Draw-a-Person Test (DAP) for evolving personality traits, and the Family Environmental Scale (FES). Statistical analysis data were tabulated in Microsoft Excel (Microsoft Corporation, Redmond, WA) and analyzed using SPSS version 16.0 (SPSS Inc., Chicago, IL). Categorical variables were summarized as frequencies and percentages, while numerical variables were expressed as means and standard deviations (SD). Inferential statistics included the chi-squared (χ^2^) test for categorical associations. Data were found to be normally distributed via the Shapiro-Wilk test (p = 0.413). Independent t-tests were done to compare numerical means between groups. Pearson’s correlation coefficient (r), Crammer's V, and Cohen’s d were calculated to quantify the magnitude of categorical variables.

Results: Depressive episode was diagnosed in 23.44% (n = 34) of children. Most cases were aged 9-12 years (n = 26, 76.5%) with a slight male preponderance (n = 18, 52.29%). Frequent symptoms included sleep disturbances (n = 28, 82.35%), irritability (n = 26, 76.47%), and somatic complaints (n = 22, 64.7%). Environmental stressors such as bullying (χ^2 ^(1, n = 68), 11.691, p = 0.001, V = 0.415) and a lack of close friends (χ^2 ^(1, n = 68), 23.611, p = 0.00, V = 0.589) were significantly more prevalent in cases. FES results indicated that depressed children lived in domestic environments characterized by lower cohesion (p = 0.032) and higher conflict (p = 0.00) and control (p = 0.00). DAP testing revealed significantly higher scores for anxiety (p = 0.00), low self-esteem (p = 0.001), and internalized aggression (p = 0.001) in the case group. Also noted was high discordance in illness perception (88%, n = 30), as parents' severity ratings did not match the child's self-report, with 64.7% (n = 22) of parents underestimating the severity.

Conclusion: Children with depression present with varied symptoms, including somatic and behavioral problems. Childhood depression is a multi-dimensional disorder that correlates with the family environment and temperament of the child. The significant parent-child perceptual gap highlights the need for child-centric assessment tools and enhanced parental awareness to ensure early diagnosis and minimize long-term social impairment.

## Introduction

Historically, it was believed that children were incapable of experiencing depressive episodes due to an immature personality structure. This paradigm shifted after the 1970 Stockholm Congress of Pedo-psychiatrists, which established that children, like adults, meet standard diagnostic criteria for mood disorders. Characterized by persistent sadness, anhedonia, and fatigue, childhood depression often involves diminished self-esteem, sleep disturbances, and suicidal ideation. However, children frequently present with "masked" symptoms, where underlying distress is overshadowed by behavioral problems and somatic complaints [1,2].

Epidemiological data reveal that while gender distribution remains equal in childhood, a female preponderance typically emerges between ages 15 and 18 years. Prevalence rates vary by context. Western studies report ranges of 0.4% to 2.5%, whereas Indian data show point prevalence between 0.1% and 6.94% in community samples, rising significantly to 3%-68% in school-based studies. Recent research indicates an increasing trend in childhood depressive disorders. In India, clinic-based studies show prevalence rising from 2% to 13.49% over two decades. Community-based surveys report point prevalence between 0.37% and 14.5%, with higher rates often linked to the use of standardized instruments like the Childhood Depression Inventory. School-based data remain sparse but suggest a prevalence of approximately 2.33% in children under 13 years of age [3-6].

Clinical presentation in children differs significantly from that of adults. Behavioral problems are common, where distress manifests as aggression, hyperactivity, refusal to go to school, withdrawal, or regressive behavior such as enuresis. While core symptoms like anhedonia and fatigue persist, children more frequently exhibit irritability instead of sadness, and functional somatic symptoms (FSS), such as unexplained headaches or abdominal pain, are noticed more commonly. Children undergoing a depressive episode are also at risk of self-harm and suicide [7]. Furthermore, co-occurring conduct problems are not uncommon and are strong predictors of poor academic performance and long-term social impairment [8].

The etiology of childhood depression is inherently multi-factorial, stemming from an interplay of biological, social, and psychological determinants. Genetic predisposition is a possible factor, with significantly higher risks observed in children of depressed parents [9]. Psychosocially, the family environment, stressful life events, and a child’s individual temperament are critical in triggering episodes [10]. Early adverse experiences, including maternal depression, parental separation, emotional neglect, and bullying, are potent predictors of chronicity and symptom severity [11]. Parenting styles also play a role, where permissive parenting correlates positively with depression, whereas authoritative and authoritarian styles show a negative correlation [12]. Interestingly, high-quality care can moderate the negative impact of maternal depression on a child’s emotional outcome [13,14]. High negative affectivity (neuroticism) is a consistent predictor of both anxiety and depression, while low positive affectivity (surgency) is specifically linked to depressive symptomatology [15]. Children with high impulsivity or low emotionality are particularly vulnerable to inconsistent discipline or parental rejection [16].

Current literature suffers from a geographical gap, with a heavy concentration on urban adolescent populations, leaving the socio-cultural nuances of younger children (below 12 years) in India underrepresented [3-6]. Methodologically, there is a scarcity of case-control studies that integrate multi-dimensional assessments, such as projective temperamental testing and structured family environmental scales, within a single framework. Furthermore, the diagnostic gap created by "masked" symptoms and the documented discordance between child self-report and parental perception remain poorly quantified. This study aimed to address these deficits by evaluating pediatric-specific symptomatology and its incidence in the study population. It also aims to study the correlation between the child's temperamental traits and family environment through a quantitative analysis. Another aim is to assess if there is any discordance with symptom severity between the child and parent.

## Materials and methods

Study design and setting

This institution-based, observational descriptive study utilized a case-control design to examine the clinical features and multi-factorial correlates of childhood depression. The study was conducted at the Child Guidance Clinic of the Department of Psychiatry at an eastern Indian hospital as part of the primary author's master's degree thesis. Data collection spanned a 12-month period (January 2019 to January 2020), following ethical clearance.

Participants and sampling

The total enumerative sampling method was employed, screening 145 children aged five to 12 years. The final study population comprised 68 participants: 34 cases and 34 controls. To control for potential confounding by developmental stage and gender, a 1:1 matching strategy was employed. Cases and controls were strictly matched based on chronological age (±6 months) and biological sex.

Cases were consecutive patients attending the Child Guidance Clinic who met the International Classification of Diseases, 10th Revision, Diagnostic Criteria for Research (ICD-10 DCR) for a depressive episode, which are consistent for all ages [17]. Inclusion required an intelligence quotient (IQ) of more than 70 and written parental consent. Controls were recruited from the pediatric outpatient department among children attending for minor, non-chronic physical ailments (e.g., common cold). They were pair-matched for age and sex, possessed an IQ of more than 70, and were screened using the Pediatric Symptom Checklist (PSC) and clinically to rule out psychiatric morbidity [18]. Exclusion criteria for both cases and controls included major neurological disorders, intellectual disabilities, other developmental disorders, or comorbid major psychiatric or medical illnesses.

Procedures and assessment tools

Following informed consent, participants underwent a semi-structured clinical interview to collect socio-demographic data and clinical history. Socio-economic status (SES) was determined using the Modified Brahm Govind (BG) Prasad’s SES Scale [19]. The Pediatric Symptom Checklist (PSC), a 35-item scale, which is reliable and well-validated, was used to screen for emotional and behavioral problems. A score of 28 or more is considered significant [18].

The Center for Epidemiological Studies Depression Scale for Children (CES-DC), a 20-item scale, was used as a self-reported measure for cases, and the same scale was also rated by parents as a proxy to assess the severity mismatch, if any, when rated self and by proxy. It is a well-validated Likert-type scale scored from 0-3 with a maximum score of 60, and scores of 15 and above being significant for depression [20].

The Draw-a-Person Test (DAP), a projective assessment, was utilized to evaluate the children's temperament and intelligence [21]. DAP was chosen as a measure as it is culturally appropriate for Indian children who may be shy or have varying levels of literacy, especially with respect to symptomatology, and may not be able to voice their concerns. Each child was given a pencil, eraser, and unlined paper and instructed to draw a person. Following the standardized administration protocol by Harris (1963) and Koppitz (1968), the examiner noted behavioral observations such as hesitations, excessive erasing, and the sequence of body parts drawn [22,23]. Scoring was conducted using a dual-modality approach. Quantitative developmental maturity was calculated using the Goodenough-Harris system, which assigns points for the presence of 73 specific anatomical details and proportions, yielding a standard score based on these drawings, where 90-109 represents the average range and scores below 70 suggest intellectual impairment. The qualitative indicators were evaluated based on the Koppitz criteria to identify temperamental vulnerabilities. Specific indicators, such as figure size, line quality, shading, and omissions, were analyzed to screen for various temperamental traits. The DAP drawings were subjected to a blinded theme analysis by at least two independent clinical raters trained in projective techniques, with discrepancies resolved through consensus.

**Figure 1:**
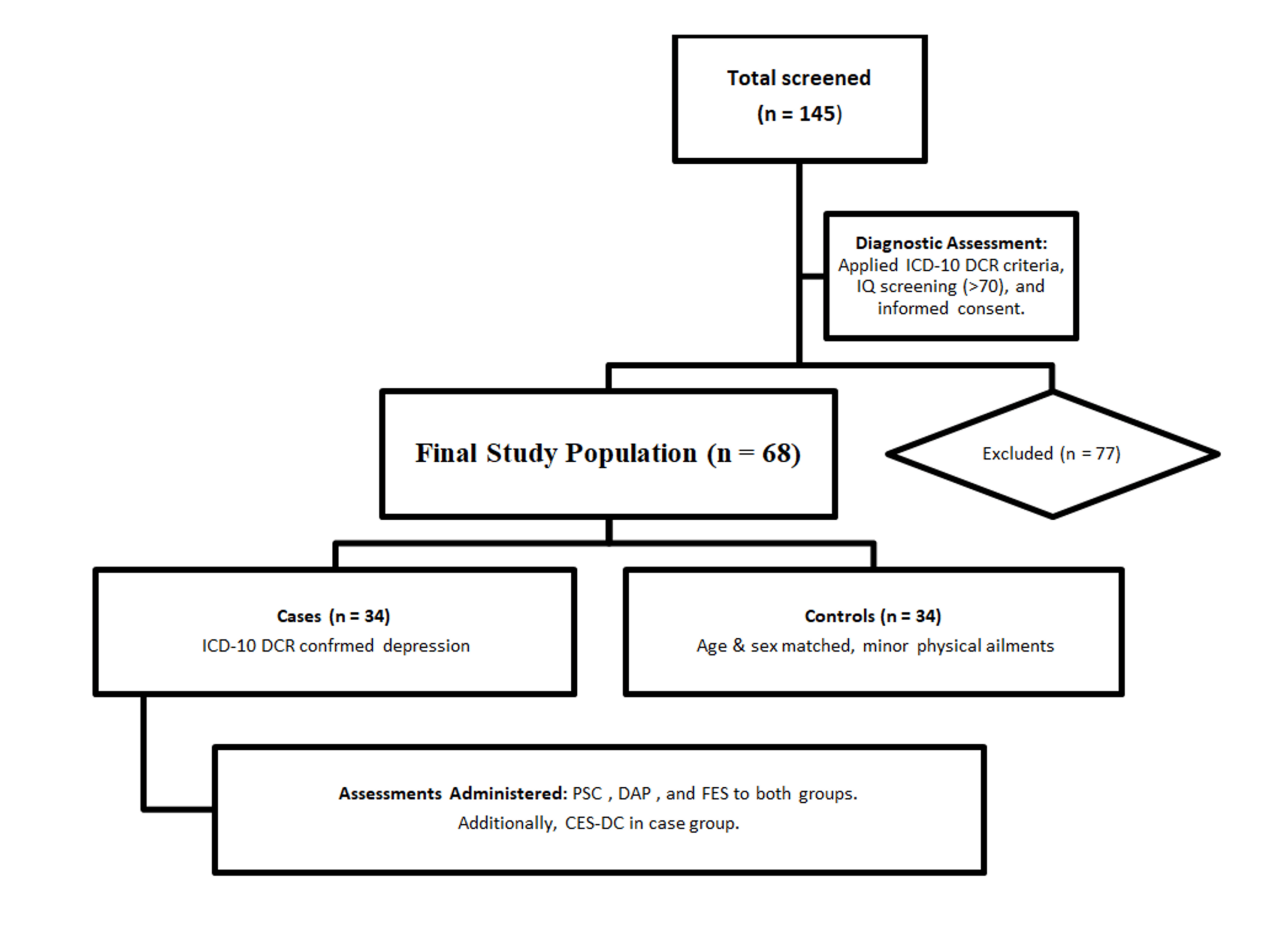
Flow of events in the study. ICD-10 DCR: International Classification of Diseases, 10th Revision, Diagnostic Criteria for Research; IQ: intelligence quotient; PSC: Pediatric Symptom Checklist; DAP: Draw-a-Person Test; FES: Family Environmental Scale; CES-DC: Center for Epidemiological Studies Depression Scale for Children.

The Family Environmental Scale (FES), a 69-item scale developed and validated in India with Likert-type scoring ranging from 1 to 5, assessed eight sub-scales across three dimensions: relationship (cohesion, expressiveness, conflict, acceptance), personal growth (independence, active-recreational orientation), and system maintenance (organization, control). Interpretation is based on the raw score obtained for each subscale. Higher scores indicate a higher prevalence of that specific trait within the family (Figure 1) [24].

For analytical purposes, CES-DC scores were treated as both continuous (means) and categorical (elevated group for scores till 35 and very elevated groups for scores above 35) to allow for further nuanced correlation. After formal, content, and thematic analysis of the DAP, recurring themes were converted to categorical variables (present/absent) and analyzed accordingly.

Statistical analysis data were tabulated in Microsoft Excel (Microsoft Corporation, Redmond, WA) and analyzed using SPSS version 16.0 (SPSS Inc., Chicago, IL). Categorical variables were summarized as frequencies and percentages, while numerical variables were expressed as means and standard deviations (SD). Inferential statistics included the chi-squared (χ^2^) test for categorical associations. Data were found to be normally distributed via the Shapiro-Wilk test (p = 0.413), justifying the use of parametric measures for further analysis. Independent t-tests were used for comparing numerical means between groups. Pearson’s correlation coefficient was calculated, and statistical significance was set at p = 0.05 with a 95% confidence interval. Crammer's V was calculated to estimate the strength of association for categorical variables. Cohen’s d was calculated for all significantly FES sub-scales to quantify the magnitude of environmental differences.

## Results

During the one-year study period at the institution, a total of 145 children were screened. Of these, 34 children met the formal ICD-10 DCR criteria for a depressive episode, yielding a point prevalence of 23.44% (n = 34) among the clinic-attending population. The final case group (n = 34) and the age- and sex-matched control group (n = 34) were analyzed across various parameters. The demographic analysis revealed that the majority of the study population (n = 26, 76.5%) was in the late childhood/early adolescent bracket of nine to 12 years, while 23.5% (n = 8) were aged five to nine years. The mean age of the study population was 10.47 years (SD = 1.34). Gender distribution within the case group showed a slight male preponderance (18 boys, 52.29%; 16 girls, 47.05%). Interestingly, while the gender ratio was equal in the five to nine years age group, boys (n = 14, 41.18%) were more frequently diagnosed in the nine to 12 years age group. Residence was predominantly rural (n = 20, 58.8%) across both groups, and no statistically significant differences were observed regarding parental education (maternal: χ^2 ^(6, n = 68) 7.486, p = 0.278, V = 0.332; paternal: χ​​​​​^2 ^(5, n = 68) 6.438, p = 0.266, V = 0.308), father's employment status (χ^2 ^(3, n = 68) 3.594, p = 0.309, V = 0.230), or maternal occupation (χ*^2 ^*(3, n = 68) 4.097, p = 0.251, V = 0.245). Socio-economic status (categorized by the modified BG Prasad scale) also showed no significant difference (χ^2 ^(3, n = 68) 0.508, p = 0.917, V = 0.86) between cases and controls.

The clinical profile of childhood depression in this study was a hybrid of core affective symptoms and "masked" behavioral or somatic manifestations. The mean CES-DC score was 24.24 with a standard deviation of 5.366. The most frequently reported symptoms were disturbed sleep (n = 28, 82.35%) and tiredness (n = 28, 82.35%). These were closely followed by irritability and loneliness (n = 26, 76.47% each). Affective symptoms like sadness and crying spells were reported by 70.58% (n = 24) of the cases. "Masked" symptoms were highly prevalent, and 70.58% (n = 24) of children exhibited behavioral problems, school refusal, and low confidence. Somatic complaints, such as persistent aches and pains, were reported by 64.7% (n = 22) of cases. Cognitive and academic impacts were also evident, with 58.82% (n = 20) reporting forgetfulness and 52.94% (n = 18) showing a decline in academic performance. Children with ongoing depressive episodes also expressed a death wish (n = 18, 52.94%) and had attempted self-harm (n = 12, 35.29%) (Table 1).

**Table 1:** Distribution of depressive symptoms in the case group.

Depressive symptoms	Frequency (%)
Disturbed sleep	28 (82.35)
Tiredness	28 (82.35)
Feeling irritable	26 (76.47)
Feeling lonely	26 (76.47)
Feeling sad	24 (70.58)
Crying	24 (70.58)
Behavioral problems	24 (70.58)
Low confidence	24 (70.58)
Refusal for school	24 (70.58)
Feeling unloved	24 (70.58)
Aches and pains	22 (64.7)
Fear of bad things happening	22 (64.7)
Poor interaction with people	22 (64.7)
Poor appetite	20 (58.82)
Forgetfulness	20 (58.82)
Feeling that things won’t work out	20 (58.82)
Lack of interest in play and leisure activities	18 (52.94)
Decline in academic performance	18 (52.94)
Death wish	18 (52.94)
Attempted self-harm	12 (35.29)
Blaming self	12 (35.29)

Significant differences emerged regarding the domestic environment. While all children in the control group lived with both parents, only 64.7% (n = 22) of the cases resided with both parents. The remaining cases lived with grandparents (n = 6, 17.6%), single mothers (n = 4, 11.7%), or step-parents (n = 2, 5.8%). Children from broken families (n = 8, 23.5%) were found exclusively in the case group. Furthermore, depressed children (n = 14, 41.18%) were significantly less likely to have siblings (χ^2 ^(1, n = 68) = 16.48, p = 0.00, V = 0.429) compared to controls (n = 30, 88.23%). Perinatal and early developmental factors were also contributory. The incidence of antenatal maternal stress (n = 20, 58.82%, χ^2 ^(1, n = 68) = 16.485, p = 0.00, V = 0.492) and low birth weight (n = 16, 47.07%, χ^2 ^(1, n = 68) = 14.809, p = 0.00, V = 0.467) was significantly higher in cases and both positively correlated with childhood depressive episode. Although pre-term delivery was also more frequent among cases, this did not reach statistical significance (χ^2 ^(1, n = 68), 0.731, p = 0.393, V = 0.104). These findings may suggest that early biological and maternal stressors may create baseline vulnerability for later depressive episodes, but need further evaluation.

The independent t-test on FES scores revealed that the case group lived in significantly more pathological domestic environments. Cohen's d was calculated only for those domains where cases and controls significantly differed, with family cohesion being statistically lower with a medium effect size, while conflict and control scores were significantly higher with a large effect size (Table 2).

**Table 2:** Comparison of scores on domains of the Family Environmental Scale between cases and controls (independent t-test). SD: standard deviation; t: t-statistic (size of difference); df: degree of freedom; S: significant; NS: not significant; CI: confidence interval; FES: Family Environmental Scale.

Domains of FES	Group	Mean	SD	t	df	p-value	Cohen’s d (effect size)	95% CI (lower)	95% CI (upper)
Cohesion	Cases	45.71	9.077	-2.19	56.27	0.032 (S)	0.531 (S)	-7.765	-0.353
	Controls	49.76	5.831						
Expressiveness	Cases	30.47	5.350	1.47	66	0.147 (NS)	-	-0.657	4.304
	Controls	28.65	4.886						
Conflict	Cases	41.29	8.611	10.27	56.47	0.00 (S)	2.489 (S)	14.537	21.581
	Controls	23.24	5.565						
Acceptance and caring	Cases	38.88	5.221	-0.73	66	0.471 (NS)	-	-3.534	1.652
	Controls	39.82	5.485						
Independence	Cases	26.82	6.571	0.27	66	0.788 (NS)	-	-2.633	3.456
	Controls	26.41	5.990						
Active recreational orientation	Cases	29.00	5.472	-1.5	66	0.138 (NS)	-	-4.521	0.639
	Controls	30.94	5.181						
Organization	Cases	7.76	1.499	-0.68	66	0.499 (NS)	-	-0.926	0.455
	Controls	8.00	1.348						
Control	Cases	16.18	2.289	8.15	66	0.00 (S)	1.976 (S)	3.86	6.37
	Controls	11.06	2.860						

Correlation analysis revealed that lower scores on domains of expressiveness, acceptance/caring, and independence correlated with higher scores on CES-DC. In children belonging to families that have lower scores on domains of expressiveness, acceptance/caring, and independence, a higher severity of depressive illness was noted (Table 3).

**Table 3:** Correlation of score on CES-DC and various domains of FES. S: significant; NS: not significant; FES: Family Environmental Scale; CES-DC: Center for Epidemiological Studies Depression Scale for Children.

Domains of FES		Cohesion	Expressiveness	Conflict	Acceptance and caring	Independence	Active recreational orientation	Organization	Control
CES-DC	Pearson correlation	-0.159	-0.407	-0.015	-0.529	-0.606	-0.109	-0.144	-0.043
	p-value	0.369 (NS)	0.017 (S)	0.934 (NS)	0.001 (S)	0.00 (S)	0.538 (NS)	0.418 (NS)	0.809 (NS)

**Figure 2:**
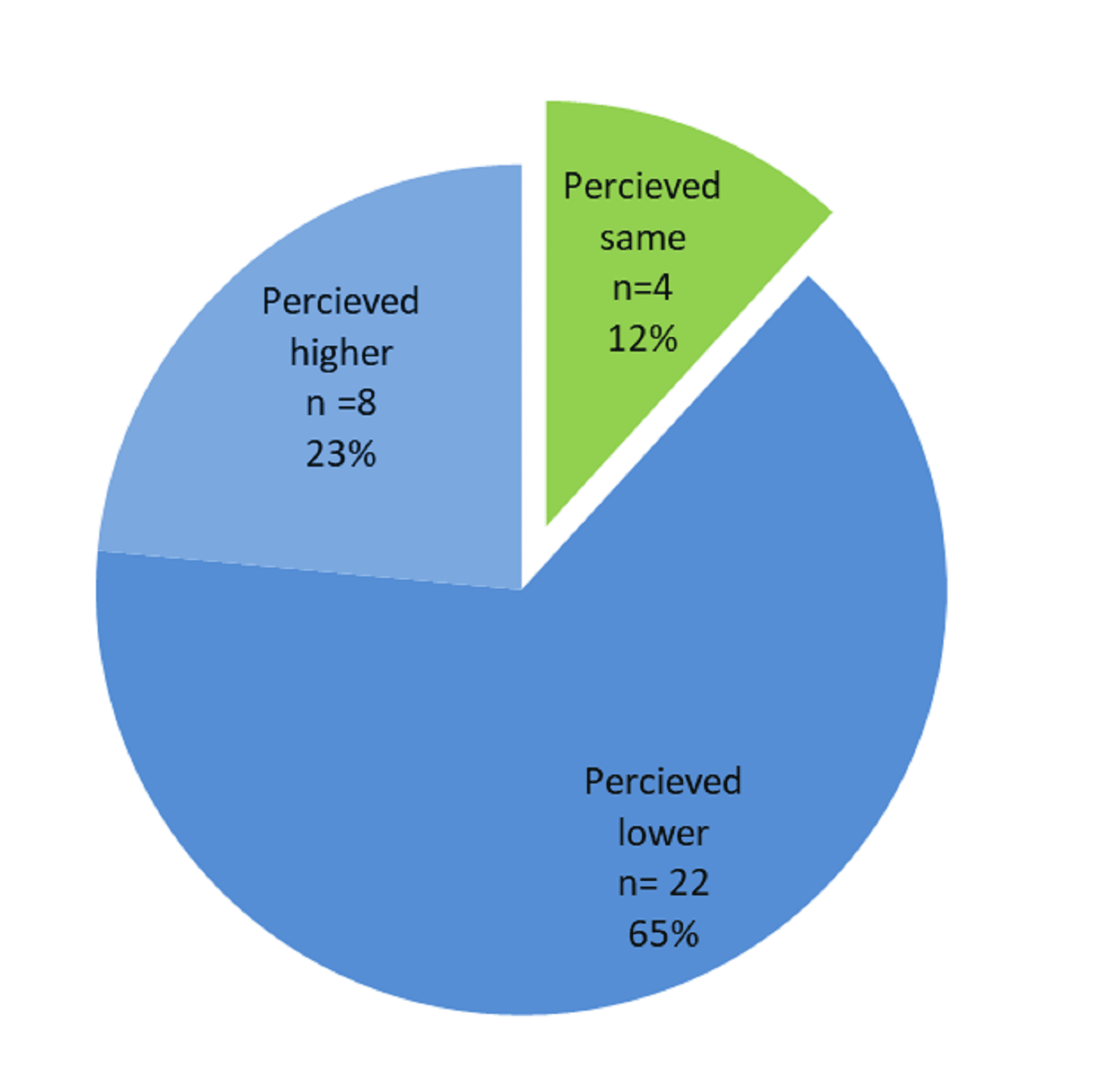
Pie chart depicting the parent-child perceptual discordance in the severity of depressive episode. n = frequency.

The CES-DC scores categorized 41.17% (n = 14) of cases in the elevated range, while 58.82% (n = 20) presented with very elevated severity scores. Girls (n = 12, 75%) were found to have significantly higher severity scores than boys (n = 8, 44.44%). There was a high level of discordance between child and parent perceptions of the illness. In 88% (n = 30) of cases, parents’ severity ratings did not coincide with the child’s self-report. Specifically, 64.7% (n = 22) of parents underestimated the severity of their child’s depression, while only 11.76%(n = 4) accurately perceived the level of distress (Figure 2).

Environmental stressors significantly differentiated the groups. Experiencing bullying at school (χ^2 ^(1, n = 68), 11.691, p = 0.001, V = 0.415) and a lack of close friends (χ​​​​​​​^2 ^(1, n = 68), 23.611, p = 0.00, V = 0.589) were statistically more prevalent in cases. Additionally, receiving critical comments from family members was a significant predictor (χ​​​​​​​^2 ^(1, n = 68), 18.133, p = 0.00, V = 0.516). Temperamental traits assessed via the DAP test, on categorical analysis, showed that cases scored significantly higher on anxiety, regression, dependency, low self-esteem, internalized aggression, withdrawal, guilt, and somatic preoccupation (Table 4).

**Table 4:** Distribution of temperament related factors between cases and controls. S: significant; NS: not significant; n: frequency.

Temperament-related factors	Cases, n (%)	Controls, n (%)	Pearson’s chi-square value	Degree of freedom	Crammer’s V	p-value
Anxiety	30 (88.23)	10 (29.41)	24.286	1	0.598	0.00 (S)
Regression	30 (88.23)	10 (29.41)	24.286	1	0.598	0.00 (S)
Dependence	28 (82.35)	18 (52.94)	6.719	1	0.314	0.019 (S)
Low self-esteem	24 (70.58)	10 (29.41)	11.529	1	0.412	0.001 (S)
Aggression (internalized)	22 (64.7)	8 (23.52)	11.691	1	0.415	0.001 (S)
Aggression (externalized)	16 (47.05)	18 (52.94)	0.235	1	0.059	0.809 (NS)
Withdrawal	22 (64.7)	8 (23.52)	11.691	1	0.415	0.001 (S)
Guilt	14 (41.11)	4 (11.76)	7.556	1	0.333	0.012 (S)
Interpersonal issues	24 (70.58)	8 (23.52)	15.111	1	0.471	0.00 (S)
Assaultive behavior	16 (47.05)	16 (47.05)	0.00	1	0.0	1 (NS)
Sensitivity	24 (70.58)	8 (23.52)	15.111	1	0.471	0.00 (S)
Poor judgment	12 (35.29)	8 (23.52)	1.133	1	0.129	0.425 (NS)
Somatic preoccupation	20 (58.82)	6 (17.64)	12.205	1	0.424	0.001 (S)
Obsessive-compulsive tendency	12 (35.29)	4 (11.76)	5.231	1	0.277	0.043 (S)
Shyness	18 (52.94)	8 (23.52)	6.227	1	0.303	0.024 (S)
Insecurity	28 (82.35)	6 (17.64)	28.471	1	0.647	0.00 (S)
Assertiveness	8 (23.52)	26 (76.47)	19.059	1	0.529	0.00 (S)
TOTAL	34 (100)	34 (100)				-

## Discussion

In this study, 23.44% (n = 34) of children attending the Child Guidance Clinic were diagnosed with a depressive episode. The majority of participants (n = 26, 76.47%) were aged nine to 12 years, with a recorded onset as young as seven years, notably lower than other studies conducted in India. The incidence of depressive episodes is higher in this study as compared to those reported in earlier studies, likely reflecting the rising trend of pediatric depression and increased parental awareness in tertiary care settings [3]. A slight male preponderance (n = 18, 53%) was observed, which may be due to boys exhibiting more externalizing behavioral problems that prompt earlier clinical consultation.

The most frequent symptoms were disturbed sleep and tiredness (n = 28, 82.35% each), while irritability (n = 26, 76.47%) was reported more often than sadness (n = 24, 70.58%). Behavioral problems and school refusal were present in over 70% (n = 24) of cases, while 35.29% (n = 12) reported attempted self-harm. These mood findings align with Indian literature, highlighting that pediatric depression predominantly presents with irritable mood and functional impairment in academic and social spheres [3]. However, sleep disturbance outnumbering the mood symptoms is a finding unique to this study. Additionally, poor body image was more common in cases, often exacerbated by critical comments from family and peers. This finding may be a result of the depressive cognition in children, where they perceive rejection more strongly from peers and family. The presence of a close friend appeared to act as a buffer, corroborating findings that social support is a critical protective factor against internalizing symptoms [25].

No statistically significant differences were found between cases and controls regarding socio-economic status, parental education, or parental employment. However, an inverse correlation between socio-economic status and depressive episode in children has been mentioned in the literature [26]. This finding is unique and suggests that while lower SES is prevalent in government hospital samples, it may not be the primary driver of depression in this specific cohort. Intact family structures appeared protective against a depressive episode. The FES revealed that cases reported significantly lower cohesion and higher control and conflict than controls. Large effect sizes were found for conflict and control. While acceptance and expressiveness did not significantly differ between cases and controls overall, they showed a significant negative correlation with depression severity. This indicates that while a lack of cohesion may trigger depression, a lack of independence and acceptance specifically drives the episode's severity [27].

A critical finding, unique to the study, is that 88% (n = 30) of parents misperceived the severity of their child's depression, with 64.7% (n = 22) underestimating it. This discrepancy may stem from the internalizing nature of the disorder or a lack of emotional closeness. This perceptual gap highlights a significant barrier to early intervention, as the majority of parents were unaware of the actual depth of their child’s suffering. Parents often only seek help when symptoms manifest as "bad behavior," failing to recognize the underlying affective distress [8]. The higher severity reported by girls may reflect societal factors where early symptoms are easily missed in girls because quiet, withdrawn behavior is more socially tolerated [2,3]. While there are studies that focus on the parental perception of a larger umbrella of mental health needs of children, there is a dearth of studies focused on such perceptual gaps with respect to depression in children [28].

Another strength of this study was the use of the DAP test, a qualitative projective tool, to assess temperament alongside the standardized, validated instruments. Unlike parent-rated questionnaires, the DAP captured internalizing traits that might not yet manifest in overt behavior, thus addressing the masking of symptoms in depressive disorder in children [23]. This study is statistically superior to simple cross-sectional surveys as the use of age- and sex-matched controls strengthens the validity of the environmental findings [29]. Assessment of temperament traits echoed the existing literature findings that more children with depression showed anxiety, regression, dependence, poor self-esteem, internalized aggression, and obsessive tendencies [21,23]. They also reported withdrawal, guilt, interpersonal issues, somatic preoccupations, shyness, and insecurity more often than the controls and lacked assertiveness [30].

Limitations include a small sample size and self-report bias. The single-center hospital setting may introduce referral bias and also limit the generalization of these results to wider populations. Additionally, the cross-sectional nature of the study precludes establishing definitive causality. In this study, as the authors were clinicians and part of the treating team, blinding for the recruitment of case-control status of the children could not be carried out; however, blinded theme analysis was carried out for DAP. Though the case and control groups did not significantly differ with respect to SES and parental education; however, these could have been possible confounders.

Further research is needed on establishing the causality of depressive episodes in childhood. It would be worthwhile to track and assess longitudinal outcomes into adolescence and adulthood. Developing and evaluating family-based psychoeducational interventions is essential to bridge the documented parent-child perception gap and foster improved long-term clinical outcomes through early detection of symptoms and addressing the family environment.

## Conclusions

This study highlights that childhood depression is a multi-dimensional disorder driven by perinatal stress, domestic environments, and evolving personality traits in children. The significant parent-child perceptual gap and the shift toward withdrawal in severe cases underscore the need for clinicians to look beyond behavioral symptoms and utilize child-centric assessment tools. Antenatal maternal care and improvement of the school and family environment can go a long way in the prevention of depressive episodes in children. If left untreated, childhood depression can lead to protracted physical and mental health problems, hampering overall growth and social development. Therefore, early diagnosis and intervention are of utmost importance to minimize adverse long-term outcomes.
